# A Comparison of Chlorhexidine-Alcohol and Povidone-Iodine-Alcohol on the Incidence of Surgical Site Infection

**DOI:** 10.7759/cureus.51901

**Published:** 2024-01-08

**Authors:** Adegbolahan Fakoya, Adefemi Afolabi, Omobolaji Ayandipo, Olufunmilola Makanjuola, Olalekan Adepoju, Oluwasanmi Ajagbe, Oludolapo O Afuwape

**Affiliations:** 1 Endocrine Surgery, University College Hospital, Ibadan, NGA; 2 Endocrine Surgery, University of Ibadan, Ibadan, NGA; 3 Oncology, University of Ibadan, Ibadan, NGA; 4 Micobiology, University College Hospital, University of Ibadan, Ibadan, NGA; 5 Surgery, National Health Service (NHS) Grampian, Elgin, GBR; 6 Surgery/Oncology, University College Hospital, Ibadan, NGA; 7 Surgery/General Surgery, University College Hospital, University of Ibadan, Ibadan, NGA

**Keywords:** comparism, incidence, ssi, povidone iodine-alcohol, chlorhexidine-alcohol

## Abstract

Background: Surgical site infection (SSI) persists as a global challenge, accounting for 20%-25% of all healthcare-associated infections. The SSI rate has been reported to range from 2.5% to 41.9%. Skin preparation with acceptable antiseptic preparations has a high recommendation from the Centers for Disease Control as an SSI preventive measure.

Aim: The aim was to compare the efficacy of 10% povidone-iodine in 70% isopropyl alcohol with 2% chlorhexidine in 70% isopropyl alcohol in preventing SSI.

Method: This prospective randomized study included patients who were followed up for 30 days looking for SSI. Swabs were taken from wounds that developed SSI. A culture of all swabs was done.

Result: One hundred and fifty-three patients were recruited into the study. Overall, eight (5.23%) of the 153 patients developed SSI. The SSI rate in clean wounds was 2.6%, while the SSI rate in clean-contaminated wounds was 7.9%. No statistically significant difference was found (p=0.141) between the two groups.

## Introduction

Surgical site infection (SSI) persists as a global challenge, accounting for 20%-25% of all healthcare-associated infections. [1] This is seen despite improved surgical practices and understanding of the principle of the use of antimicrobials. The rate of SSI has been reported to range from 2.5% to 41.9% [2].

Surgical wound infection causes a considerable economic burden to the health care facility, the surgeon, and the patient [3].

The patient has been identified as an endogenous reservoir for infection, especially SSI, with the skin flora implicated as a main pool of pathogens. The skin flora comprises both resident and transient organisms. *Staphylococcus aureus*, a major commensal of the skin, is the most common isolate from post-operative wound infection [4,5].

One of the recommendations of the Centers for Disease Control (CDC) as a preventive measure for SSI is preoperative skin preparation. Even though the skin cannot be sterilized because approximately 20% of the resident flora are beyond the reach of surgical scrubs and antiseptics, skin preparation reduces the bacteria load significantly to reduce infection rates. Hitherto no specific antiseptic agent has been considered the first choice because no study has adequately assessed the comparative effects of preoperative skin antiseptics in a well-controlled operation-specific study [6].

The aim of this study, therefore, was to compare the effect of a “two-phase scrub” of 10% povidone iodine followed by 70% isopropyl alcohol and 2% chlorhexidine in 70% isopropyl alcohol on SSI in clean and clean-contaminated wounds in general surgical practice.

## Materials and methods

Patients from 14 years of age who underwent clean and clean-contaminated surgical procedures were included in this study. Patients with evidence of infection at or adjacent to the operation site were excluded from this study. Ethical approval was obtained from the joint University of Ibadan/University College Hospital Ethical Committee. Informed consent for the study was obtained from the appropriate person, which included either the patient or a parent/guardian.

All the patients whose surgical wound type was pre-adjudged to be either clean or clean-contaminated within the study period were recruited for the study. A random allocation of antiseptic protocol to the pre-adjudged wound type was done. Two separate bags were allotted to each wound group and labeled accordingly. Each bag contained 80 tags, with each half bearing an identity either for chlorhexidine/alcohol or povidone iodine/alcohol antiseptic protocol. The patient picked from the bag allotted to his/her group, thereby deciding the antiseptic protocol to be used. 

The patients were recruited through the emergency department and wards.

Skin preparations were done by residents who had been trained on the appropriate skin preparation technique, especially the use of chlorhexidine in alcohol applicator. Skin preparation was done using an applicator containing 2% chlorhexidine in 70% isopropyl alcohol as a paint or 10% povidone iodine paint using a swab on a holder, followed by alcohol cleanse using a swab on a holder. Skin washing to remove dirt was done with Savlon (an antiseptic solution containing 0.3% cetrimide and 0.03% chlorhexidine) prior to the application of the randomly allotted skin preparation antiseptics.

The patients were followed up within a 30-day interval post-surgery. At discharge, the patients were educated on the symptoms and signs of a SSI and were instructed to present to the ward as soon as they noticed any features that suggested a SSI. A wound swab for culture was taken from the wound site at the diagnosis of a surgical site infection.

 A data collection form (Appendix II) was used to capture data, which were analyzed using IBM SPSS Statistics for Windows, Version 20.0 (Released 2011; IBM Corp., Armonk, New York, United States). Patients’ demographic characteristics, co-morbidities, social and drug history, American Society of Anesthesiologists (ASA) classification, cadre of surgeons, skin antiseptic protocol, operative details, and outcomes were compared using chi-square. Logistic regression analysis was used to compare significant independent risk factors and primary outcome-the occurrence of SSI. Pearson’s correlation coefficient was used to further assess positive and negative associations. The level of statistical significance was taken as P < 0.05.

## Results

**Figure 1:**
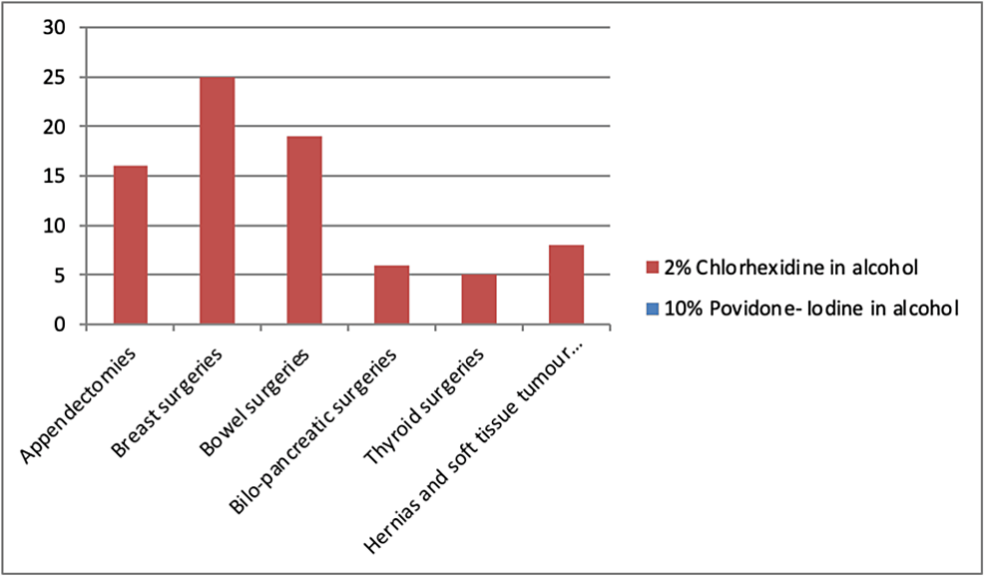
Numerical distribution of 153 patients across six categories of surgical procedures as randomized into the two skin preparation protocols.

One hundred and fifty-three patients were recruited into the study. The ages of participants ranged between 14 and 83 years, with a mean age of 41.39 ± 18.02 years. Three-fifths were female (60.8%). Figure 1 shows how these were distributed across the categories of surgical procedures.

Body mass index, alcohol consumption, smoking, and intercurrent medical illnesses did not have a statistically significant influence on the occurrence of SSI. Also, neither the class of surgery nor the cadre of surgeons appeared to significantly affect the development of SSI (Table 1).

**Table 1:** Clinicopathological and intraoperative parameters influencing the development of SSI. DM: diabetes mellitus; ASA: American Society of Anesthesiologists; SSI: surgical site infection. P-values are based on chi-square.

Parameter	Number	Percentage (%)	P value
Body mass index			0.84
Underweight	10	6.5	
Normal	83	54.2	
Overweight	44	28.8	
Obese	16	10.5	
Co-morbidities			0.82
Hypertension	29	19	
DM	6	3.9	
Hypertension and DM	2	1.3	
Others (peptic ulcer, asthma)	8	5.2	
Alcohol consumption			0.615
No	125	81.7	
Yes	28	18.3	
Smoking			0.064
Yes	12	7.8	
No	141	92.2	
Duration of surgery			0.236
<1 hour	39	25.5	
1-2 hours	85	55.5	
>2 hours	29	19	
Class of surgery			0.225
Elective	106	69.3	
Emergency	47	30.7	
ASA classification			0.008
I	73	47.7	
II	59	38.6	
III	19	12.4	
IV	2	1.3	
Cadre of surgeons			0.469
Senior registrar	95	62.1	
Consultant	58	37.9	

However, the ASA classification was found to be of statistical importance (P = 0.008). After adjusting for the duration of the procedure and whether surgery was elective or emergency, patients with a higher ASA class remained statistically significantly more likely to develop SSI, with subjects in ASA class III about 11 times more likely to develop SSI (OR at 95% CI = 1.138-110.470; P = 0.038).

Irrespective of the wound type, cadre of surgeons, and skin preparation protocol used, insertion of a wound drain was found to have a negative association (albeit weak) with the development of SSI (OR at 95% CI = 0.026-0.818; P = 0.029). This implies that SSI is less likely to develop when a wound drain is used (Pearson’s correlation coefficient = -0.189; P = 0.021). Seventy-five of the 153 patients were randomized into the 10% povidone-iodine/alcohol group and 78 into the 2% chlorhexidine in alcohol group (Table 2).

**Table 2:** Antiseptic protocol for the wound types (n = 153).

		Antiseptic protocol		Total
		10% Povidone-iodine in alcohol	2% Chlorhexidine in alcohol	
Wound type	Clean	38	39	77
	Clean-contaminated	37	39	76
Total		75	78	153

Eight (5.23%) of the 153 patients developed SSI: two of the 77 clean wounds and six of the 76 clean-contaminated wounds. This implies that the SSI rate of clean wounds was 2.6%, while the SSI rate of clean-contaminated wounds was 7.9% (Table 3).

**Table 3:** SSI in the respective wound types (n = 153). SSI: surgical site infection.

		Was the incision site infected?			Total
		No	Yes, superficial incisional	Yes, organ space	
Wound type	Clean	75	0	2 (2.6%)	77
	Clean-contaminated	70	6 (7.9%)	0	76
Total		145	6	2	153

There was a higher percentage of SSI in the povidone-iodine group (six out of 75) compared to the chlorhexidine group (two out of 78), 8.0% and 2.6%, respectively; however, this was also not found to be statistically significant (P = 0.131) (Table 4).

**Table 4:** Comparison of SSI occurrence based on wound type and stratified into the skin preparation protocol groups (n = 153) using chi-square. SSI: surgical site infection.

	Antiseptic protocol		SSI per group (%)	P-value
	10% Povidone-iodine in alcohol	2% Chlorhexidine in alcohol		
Clean wounds with SSI Yes No	2 36	0 39	2.6	0.152
Clean-contaminated wounds with SSI Yes No	4 33	2 37	7.9	0.358

## Discussion

SSI remains a notable complication following surgical interventions. It is a preventable outcome if evidence-based strategies are appropriated [7]. The CDC guidelines for the prevention of SSI 2017 comprise a number of recommendations on modalities for preventing SSIs. Pre-operative skin preparation with an alcohol-based antiseptic agent has a top-ranking recommendation based on high-quality clinical evidence [7]. The efficacy of two widely accepted antiseptic agents was determined. 2% Chlorhexidine in 70% isopropyl alcohol and 10% povidone-iodine with 70% isopropyl alcohol were compared with the primary outcome being the rate of SSI [8].

The estimated SSI rates in clean and clean-contaminated wounds are 1%-5% and 3-11, respectively. The observed SSI rate in this study for this same group of wounds fell within these values: 2.6% for the clean wounds and 7.9% for the clean-contaminated wounds.

The need for skin preparation cannot be overemphasized. The skin has been noted as a major reservoir of endogenous organisms contributing to SSI [5,9]. These pools of skin microbiota are polymicrobial, both resident and transient. A particular choice of antibiotics may be grossly inadequate for the prevention of SSI, so there is a need for on-site reduction of bacterial load. The role of antibiotics in patients with these classes of surgical wounds is prophylactic, and their use should not extend beyond the perioperative period.

On the isolation of the data comparing the wound type with the antiseptic protocol, chlorhexidine in alcohol was not found to have superior efficacy over povidone-iodine with alcohol both in the clean and clean-contaminated groups. Even though the number of patients who developed SSI was higher in the povidone-iodine group, there was no significant statistical difference in the primary outcome. Studies on skin antisepsis in the past decades have used quantitative culture results as endpoints. Skin surface microbial counts were obtained before and after the application of skin preparation solutions [10].

Ostrander et al. compared the efficacy of 0.7% iodine and 74% isopropyl alcohol, 3% chloroxylenol, and 2% chlorhexidine in 70% isopropyl alcohol [11]. The latter was found to be the most effective agent for bacterial elimination. Abdeyazdan et al. tested 10% povidone-iodine and 2% chlorhexidine solution on the bacterial flora among hospitalized infants. The study demonstrated that the use of 10% povidone-iodine resulted in a significantly reduced skin pathogen [12]. However, this cannot be extrapolated to mean a reduction in SSI rates, and there are no studies to validate this assertion [10].

A recent trial compared chlorhexidine in alcohol with PI-aqueous in 849 patients who had clean-contaminated surgery across surgical specialties, with the endpoint being SSI. It was reported that the chlorhexidine solution was more effective in terms of SSI prevention for superficial incisional infection: 9.5% developed an SSI in the chlorhexidine group compared to 16.1% in the PI group [13].

The outcome of our study is similar to that of two previous studies: Rodriquez et al. [14] had a similar wound stratification, while Parks et al. [15] considered this efficacy only in clean-contaminated wounds. Neither of these studies reported the superiority of one of these antiseptic agents over the other. A prospective study of three skin preparation protocols also disproved the superiority of chlorhexidine in reducing SSI [10].

A recent systematic review and meta-analysis on the clinical efficacy and perceived role of chlorhexidine in skin asepsis revealed good evidence favoring chlorhexidine-alcohol combinations over aqueous povidone-iodine. This stance was not found sustainable against iodophors combined with alcohol [16].

SSI that ensued included both incisional and organ space. The incisional specifically the superficial subclass accounted for approximately 60% of the SSI rate. This agrees with earlier documentation of the CDC NNIS system [6]. Among the considered risk factors for SSI infections, the ASA classification and use of wound drains were found to have a significant association with the occurrence of SSI. The ASA grading remained significant even after adjusting for other risk factors. There was still a high risk of the occurrence of SSI with subjects in ASA grade III having 11 times the risk. Wound drains have been implicated in the development of SSI, especially post-mastectomy [17,18]. However, on the contrary, this study found the use of wound drains to have a negative association, though weak, with the occurrence of SSI. This shows that the use of drains reduced the risk of SSI. Approximately half of the patients who had a wound drain inserted were post-mastectomy. Seroma, with the likelihood of it becoming infected, had most likely been abated in these patients. This may account for this finding. 

In this study, our protocol of two-pronged non-proprietary 10% povidone-iodine with alcohol was compared favorably with a proprietary preparation of chlorhexidine in alcohol. Their efficacy in preventing SSI was similar.

## Conclusions

Skin antisepsis remains an outstanding SSI preventive measure. The appropriateness of the choice of antiseptics always gives a satisfactory outcome. In this study, our protocol of two-pronged non-proprietary 10% povidone-iodine with alcohol was compared favorably with a much more expensive proprietary preparation of chlorhexidine in alcohol (Chloraprep).

Therefore, there is no difference in the efficacy between 10% povidone-iodine with 70% isopropyl alcohol and 2% chlorhexidine in 70% isopropyl alcohol in preventing SSI. The synergistic effect of alcohol in combination with either chlorhexidine or povidone-iodine cannot be downplayed. The microbiology of SSI is mainly hinged on the endogenous microflora. Proper adherence to skin antisepsis and other SSI preventive measures will yield a rewarding outcome.
